# The percentage of high‐grade prostatic adenocarcinoma in prostate biopsies significantly improves on Grade Groups in the prediction of prostate cancer death

**DOI:** 10.1111/his.13888

**Published:** 2019-08-13

**Authors:** Daniel M Berney, Luis Beltran, Holly Sandu, Geraldine Soosay, Henrik Møller, Peter Scardino, Jacqueline Murphy, Amar Ahmad, Jack Cuzick, the Transatlantic Prostate Group

**Affiliations:** ^1^ Department of Molecular Oncology Barts Cancer Institute London UK; ^2^ UK Centre for Cancer Prevention, Wolfson Institute of Preventive Medicine Queen Mary University of London London UK; ^3^ Department of Pathology Queen’s Hospital, Romford London UK; ^4^ School of Cancer and Pharmaceutical Sciences King’s College London London UK; ^5^ Department of Urology Memorial Sloan‐Kettering Cancer Center New York NY USA

**Keywords:** Gleason, Grade Group, high grade, percentage, prostate cancer

## Abstract

**Aims:**

It has been recommended that the percentage of high‐grade (HG) Gleason patterns 4 and 5 should be quantified in prostate cancer. However, this has not been assessed in a cohort using prostate cancer death as an outcome, and there is debate as to whether the biopsy with the ‘worst’ percentage of HG disease or an ‘overall’ percentage of HG disease should be reported. Such data may assist in active surveillance decisions.

**Methods and results:**

Men with clinically localised prostate cancer diagnosed by needle biopsy from 1990 to 2003 were included. The endpoint was prostate cancer death. Clinical variables included Gleason score (GS), prostate‐specific antigen level, age, clinical stage, and disease extent. Deaths were divided into those from prostate cancer and those from other causes, according to World Health Organization criteria. Nine hundred and eighty‐eight biopsy cases were centrally reviewed according to criteria agreed at the Chicago International Society of Urological Pathology conference in 2014. Cores were given individual GSs and Grade Groups (GGs), and a percentage of each grade was given for each core. Both the worst percentage of HG disease seen in a biopsy series and overall percentage of HG disease were calculated. The overall percentage of HG disease was highly significant, with a hazard ratio of 4.45 for the interquartile range (95% confidence interval 3.30–6.01, *P* < 2.2 × 10^−16^), and was similar to the percentage of HG disease seen in the worst core. In multivariate analysis, both were highly significant. GG2 cases with ≤5% Gleason pattern 4 showed similar survival to GG1 cases.

**Conclusions:**

These data validate the use of percentage of HG disease to predict prostate cancer death. As both worst and overall percentage of HG disease are powerful predictors of outcome, either could be chosen to provide prognostic information.

## Introduction

Gleason grading of prostate cancer has been established for >40 years.^1^ Although the basic grading categories have remained unchanged during this time, there have been numerous changes in the methodologies used to determine the Gleason score (GS) of prostate cancer over that period. At the 2014 International Society of Urological Pathology (ISUP) Chicago conference, the concept of Grade Groups (GGs) was accepted by a broad range of clinicians and pathologists, stratifying Gleason patterns into five separate prognostic GGs.^2^, ^3^ These groupings have been validated in large radical prostatectomy cohorts, in biopsy‐based cohorts treated with radiation, and also in a cohort of conservatively treated prostate cancers.^4^

However, at this meeting there was also a consultation on the reporting of the percentage of high‐grade (HG) disease and the assignment of a GG. The reporting of HG disease was first suggested >20 years ago,^5^ and a number of other articles have suggested that it is of clinical use in predicting biochemical relapse after radical prostatectomy^6^, ^7^, ^8^ or in predicting prostate cancer death after transurethral resection of the prostate (TURP).^9^ Recently, an exceptionally large cohort of 12 823 consecutive patients and of 2971 matched preoperative biopsies was used to validate the importance of assigning the percentage of Gleason pattern 4 and 5 with biochemical relapse.^10^ This has been validated in a number of other studies,^11^, ^12^ and it has been suggested, for instance, that the percentage of grade 4 may be used to determine criteria for active surveillance in those with minimal grade 4 disease.^13^, ^14^

However, there has been no study examining the utility of assessing the percentages of Gleason patterns 4 and 5 in prostate biopsies and their correlation with prostate cancer death.

The assignation of a GS may have considerable effects on patient management, and there is significant intraobserver variation in some areas.^15^, ^16^ In most centres, active surveillance may be given only to patients with GG1 tumours. However, GG2 includes a spectrum of disease, from patients with minimal HG tumour, to those in which up to 49% is HG. It is possible that many of those patients with a small amount of Gleason pattern 4 may be also managed more conservatively.

A potential problem with assessing HG disease is whether to measure the percentage of HG disease on the ‘worst’ core or whether to make an ‘overall’ assessment of the percentage based on the cancer in all cores.

In this study, we examined the risk of prostate cancer death relative to the percentages of both Gleason pattern 4 and Gleason pattern 5 in a biopsy series treated conservatively and previously validated for the GGs.

We hypothesised that calculation of the percentage of HG disease could add prognostic information beyond that provided by standard clinicopathological parameters.

## Materials and methods

### Patients

Cases of prostate cancer were identified from three cancer registries in Great Britain. Within each region, collaborating hospitals were sought, and cases from these hospitals were reviewed. Men were included in this study if they were aged <76 years at the date of diagnosis and had clinically localised prostate cancer diagnosed by needle biopsy in 1990–2003 inclusive. The median date of diagnosis was May 2002. Patients treated with radical prostatectomy or radiation therapy within 6 months of diagnosis were excluded. This was a practical measure to ensure that all men were not initially treated with radical therapy. Only initial hormone therapy was permitted. In addition, those with objective evidence of metastatic disease (by bone scan, X‐ray, radiograph, computed tomography scan, magnetic resonance imaging, bone biopsy, lymph node biopsy, or pelvic lymph node dissection) or clinical indications of metastatic disease (including pathological fracture, soft tissue metastases, spinal compression, or bone pain), or a prostate‐specific antigen (PSA) measurement of >100 ng/ml at or within 6 months of diagnosis, were also excluded. Men who had received hormone therapy prior to the diagnostic biopsy were also excluded, because of the influence of hormone treatment on Gleason pattern. We also excluded men who died within 6 months of diagnosis or had <6 months of follow‐up.

Original histological specimens from the diagnostic procedure were requested and centrally reviewed by a panel of three expert urological pathologists to confirm the diagnosis of adenocarcinoma and to reassign GSs by using a contemporary and consistent interpretation of the Gleason scoring system.^17^, ^18^ For each core in every case, when cancer was present, percentages of Gleason pattern 3, Gleason pattern 4 and Gleason pattern 5 were given, and a total tumour length was given for each core. Stromal gaps were not deducted from the total length measurements. An overall GS was given on the basis of the whole series, and the worst GS seen in a core as published previously.^4^

The panel met and discussed all controversial cases and a selection of others to audit the dataset. In keeping with the ISUP Chicago conference and the grading in World Health Organization (WHO) 2016, cribriform and glomeruloid glands were all assigned Gleason pattern 4. Follow‐up was conducted by use of the cancer registries, and the cut‐off date was 31 December 2012. Deaths were divided into those from prostate cancer and those from other causes, according to WHO standardised criteria (WHO, 2010). National ethics approval was obtained from the Northern Multicentre Research Ethics Committee, and local ethics committee approval was obtained at each of the collaborating hospitals.

### Statistical analysis

Survival analysis was performed with a Cox proportional hazards model. The primary endpoint was time to death from prostate cancer. All events were used for estimation of hazard ratios (HRs), and observations were censored on the date of last follow‐up or death from other causes. Covariates included in the statistical analysis were GGs by worst and overall grade, the percentage of HG disease overall and in the worst core, baseline PSA level, extent of disease calculated from the percentage of positive cores, T stage, hormone treatment, and patient age. The baseline PSA level was defined as the last prediagnostic PSA measurement within 6 months before diagnosis. If no such PSA value was available, we took the first postdiagnostic PSA level within 6 months; failing that, the prediagnostic PSA level measured closest to the date of diagnosis was used. All PSA values after treatment with hormones or orchiectomy or within 3 weeks after a surgical procedure on the prostate were excluded.

The PSA level was modelled as the natural logarithm of [1 + PSA (ng/ml)]. Patients with values of >100 ng/ml were excluded as likely to have metastatic disease.

Missing PSA values were imputed by use of a median regression with GS, age and extent of disease as predictors, and PSA as outcome. Missing T stage values were imputed using the median clinical T stage among all patients. A multivariate Cox proportional hazard model applied performed with overall GG, worst GG, overall percentage of HG disease, worst core percentage of HG disease, baseline PSA level, extent of disease, T stage, hormone treatment and patient age as predictors. The primary event of interest was time to death from prostate cancer. A stepwise model selection was performed.

Spearman’s rank correlation was estimated between all variables. All applied tests were two‐sided, and *P*‐values of <0.05 were accepted as statistically significant. No *P*‐value adjustment was performed for multiple comparisons. Statistical analyses were performed with r (R Core Team (2018). R: A language and environment for statistical computing. R Foundation for Statistical Computing, Vienna, Austria. http://www.R-project.org/).

Observations were censored on the date of last follow‐up, or death from other causes. All events were used for estimation of HRs (maximum follow‐up 232 months), but follow‐up was censored at 10 years for prediction of 10‐year risks. HRs were calculated for the interquartile range (IQR), as this is a better method of comparison when the units of the different variables are very different.

Analysis was repeated with exclusion of cases with Gleason pattern 5 and examination of only the percentage of Gleason pattern 4 in GG2 and GG3 for both overall and worst models.

## Results

Six thousand five hundred and one cores from 988 individual cases were evaluated. One hundred and sixty‐nine patients (17.1% of the cohort) died from prostate cancer. The mean, median and IQR of patient age, number of cores sampled, serum PSA level and percentage of cores involved, T stage, hormonal treatment and the overall and worst percentage of HG disease are shown in Table 1.

**Table 1:** Candidate diagnostic factors in the derivation and validation datasets (*N* = 988)

Variable name	Variable	Definition/units	Median (IQR), or *n* (%)
Patient age	Age	Years	70.90 (66.69–73.65)
Overall GG	GG1	GS of 3 + 3 = 6	307 (31.1)
	GG2	GS of 3 + 4 = 7	303 (30.7)
	GG3	GS of 4 + 3 = 7	210 (21.3)
	GG4	GS of 8	56 (5.7)
	GG5	GS of 9 or 10	112 (11.3)
Worst GG	WGG1	–	307 (31.1)
	WGG2	–	244 (24.7)
	WGG3	–	206 (20.9)
	WGG4	–	111 (11.2)
	WGG5	–	120 (12.1)
PSA	PSA*	ng/ml	14.3 (8.1–31.0)
Extent of disease	Extent	% of positive cores	5.0 (2.5–8.3)
Clinical T stage	Stage†	cT1–cT2	842 (85.2)
		cT3 or higher	146 (14.8)
Hormone treatment	Hormones	Yes	574 (58.1)
Overall high‐grade disease (%)	ovHG100	–	26.2 (0.0, 70.0)
Worst core high‐grade disease (%)	HGwPC100	–	40.0 (0.0–90.0)

GG, Grade Group; GS, Gleason score; IQR, interquartile range; PSA, prostate‐specific antigen; WGG, worst Grade Group.

* Missing PSA values (*n* = 3) were imputed by use of a median regression with GS, age and extent of disease as predictors, and PSA as an outcome.

† Missing T stage values (*n* = 230) were imputed by median of observed values T1–T2 stage.

For the overall core calculation, the HR for the IQR in overall HG disease was 4.45 [95% confidence interval (CI) 3.30–6.01; *P* < 2.2 × 10^−16^]. Harrel’s C‐statistic was 0.733. This was extremely similar to the results obtained when the percentage of HG disease in the worst core was used (HR = 6.34; 95% CI 4.29–9.39; *P* < 2 × 10^−16^). Harrel’s‐C statistic was 0.727. The IQR HRs are shown in Table 2.

**Table 2:** Univariate Cox proportional hazard regression model (*N* = 988, no. of deaths = 169)

Predictor	IQR HR (95% CI)	LR $\chi_{1}^{2}$	*P*‐value	C‐index
Overall grade group (linear)	3.06 (2.48–3.78)	102.842	<2.2 × 10^−16^	0.732
Worst grade group (linear)	3.03 (2.44–3.77)	101.322	<2.2 × 10^−16^	0.730
Overall high grade (%)	4.45 (3.30–6.01)	100.709	<2.2 × 10^−16^	0.733
Worst core high grade (%)	6.34 (4.29–9.39)	99.866	<2.2 × 10^−16^	0.727
Extent of disease (% positive cores)	3.73 (2.75–5.04)	78.437	<2.2 × 10^−16^	0.704
Log(1 + PSA)	2.52 (2.00–3.18)	61.152	5.33 × 10^−15^	0.686
Stage (stage = 3)	3.70 (2.68–5.12)	51.815	6.10 × 10^−13^	0.612
Hormones (yes)	3.28 (2.26–4.74)	47.616	5.18 × 10^−12^	0.638
Age	1.21 (0.98–1.51)	3.165	0.075	0.527

C‐index, Harrell’s C‐index; CI, confidence interval; HR, hazard ratio; IQR, interquartile range; LR$\chi_{1}^{2}$, likelihood ratio test (d.f. = 1); PSA, prostate‐specific antigen.

A multivariate model was constructed by the use of backward selection, starting with a candidate set of variables consisting of all nine predictors (the ‘full model’), and with the Wald chi‐square *P*‐values of the individual factors, with a significance level of 0.05 as a rule for staying in the final model (Table 3). In the final reduced model, the overall percentage of HG disease outperformed the other variables (HR = 2.84, 95% CI 2.00–4.03), followed by extent of disease, clinical stage of disease, and serum PSA level. A further analysis comparing the final model with and without overall percentage of HG disease confirmed that this predictor contributed significantly to the model fit (*P* < 0.001).

**Table 3:** Multivariate Cox proportional hazard regression model (*N* = 988, no. of deaths = 169)

Predictor	Full multivariable model (all nine predictors)		Final model*	
	IQR HR (95% CI)	Wald $\chi_{1}^{2}$ (*P*‐value)	IQR HR (95% CI)	Wald $\chi_{1}^{2}$ (*P*‐value)
Overall grade groups (linear)	1.70 (0.61–4.74)	1.035 (0.309)	–	–
Worst grade group (linear)	0.72 (0.21–2.43)	0.288 (0.592)	–	–
Overall high grade (%)	1.89 (0.70–5.10)	1.571 (0.210)	2.84 (2.00–4.03)	34.371 (4.5 × 10^–9^)
Worst core high grade (%)	1.21 (0.26–5.61)	0.056 (0.813)	–	–
Extent of disease (% of positive cores)	1.65 (1.14–2.40)	6.975 (0.008)	1.81 (1.26–2.58)	10.595 (0.001)
Log(1 + PSA)	1.30 (0.98–1.72)	3.412 (0.065)	1.36 (1.04–1.79)	4.948 (0.026)
Stage (stage = 3)	1.52 (1.06–2.17)	5.220 (0.022)	1.58 (1.11–2.26)	6.300 (0.012)
Hormones (yes)	1.40 (0.92–2.14)	2.498 (0.114)	–	–
Age	1.06 (0.85–1.31)	0.268 (0.605)	–	–
LR $\chi_{1}^{2}$ (d.f., *P*‐value)	147.318 (9, <2.2 × 10^−16^)		142.6 (4, <2.2 × 10^−16^)	
Harrell’s C‐index (95% CI)	0.771 (0.724–0.817)		0.767 (0.721–0.814)	

Wald $\chi_{1}^{2}$ (d.f. = 1) for each model coefficient.

CI, confidence interval; HR, hazard ratio; IQR, interquartile range; LR$\chi_{1}^{2}$, likelihood ratio test; PSA, prostate‐specific antigen.

* The final model was selected by the use of backward variable selection with Wald chi‐square *P*‐values of individual factors and with a 5% significance level rule for staying in the final model. The same model was selected by forward stepwise variable selection.

**Figure 1:**
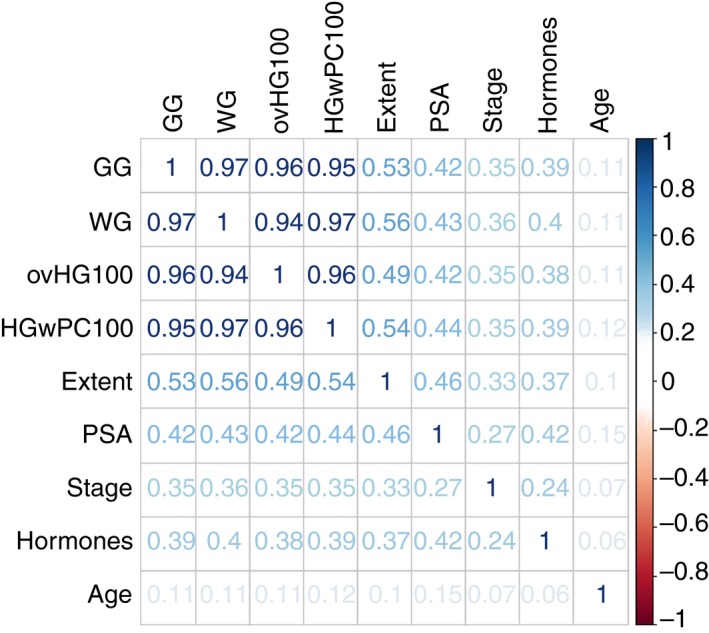
Spearman’s correlation between all nine fitted predictors from the full Cox proportional hazard regression model. GG, Grade Group; HGwPC100, worst core high‐grade disease (%); (%)ovHG100, overall high‐grade (%) disease; WG, worst grade.

A Spearman’s correlation between all nine fitted predictors in the full Cox proportional hazard model showed a very strong correlation between all of the different pathological measures of grade, but a weak correlation between age at diagnosis and all other predictors (Figure 1).

**Figure 2:**
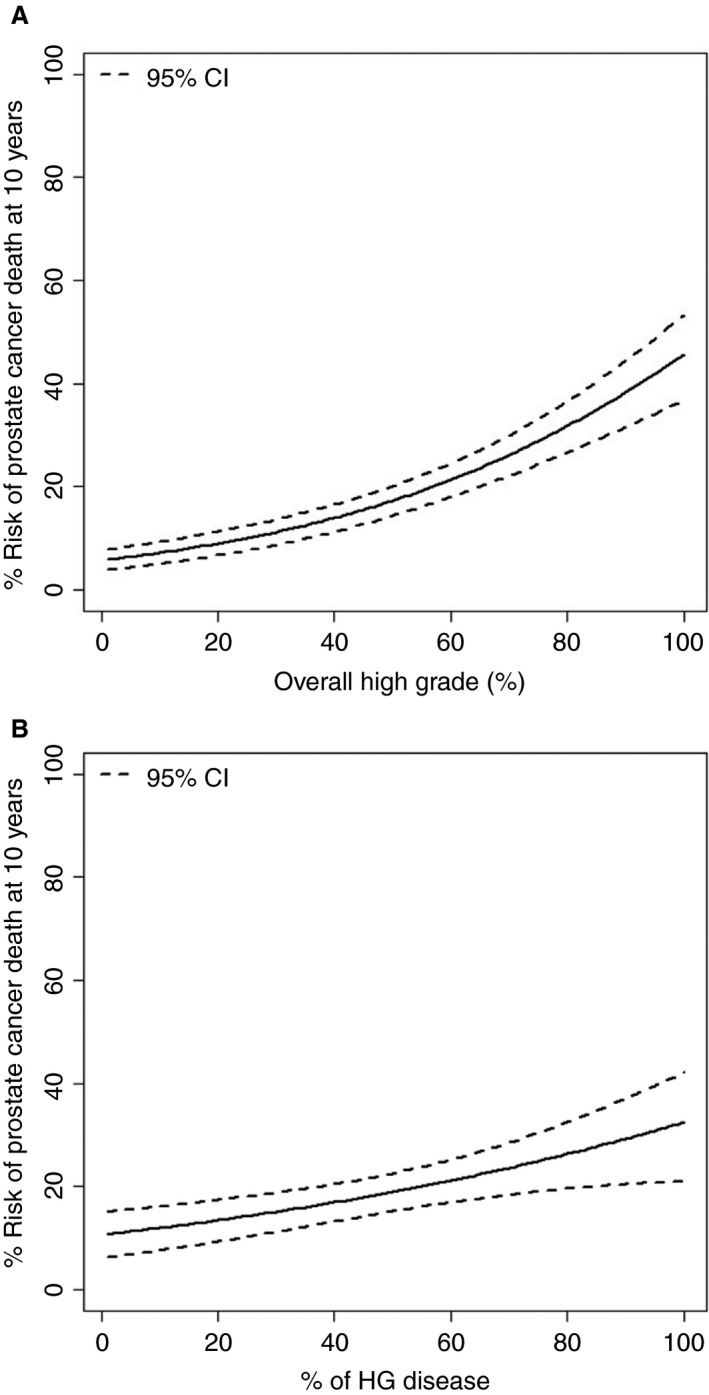
**A,** Percentage of high‐grade disease overall versus 10‐year survival. **B,** Percentage of Gleason pattern 4 overall in overall Grade Groups 2 and 3 versus 10‐year survival.

The repeat of the analysis on GG2 and GG3 alone showed that the overall percentage of Gleason pattern 4 disease remained a significant predictor of prostate cancer death in the multivariate model (Table 4). The risk of prostate cancer death in the whole cohort and limited to GG2 and GG3 is shown in Figure 2A,B.

**Table 4:** Univariate and multivariate Cox proportional hazard regression model in Grade Groups 2 and 3 (*N* = 513, no. of deaths = 91)

Predictor	Univariate analyses				Final multivariate model		
	IQR HR (95% CI)	LR $\chi_{1}^{2}$ test	*P*‐value	C‐index (95% CI)	IQR HR (95% CI)	Δ$\chi_{1}^{2}$	*P*‐value
T‐stage	2.749 (1.729–4.370)	15.463	8.41 × 10^−5^	0.581 (0.544–0.619)	1.915 (1.164–3.151)	15.463	8.41 × 10^−5^
Extent of disease (% positive cores)	2.090 (1.426–3.063)	14.985	0.0001	0.634 (0.570–0.698)	1.698 (1.142–2.525)	7.942	0.0048
Worst core high grade (%)	1.929 (1.327–2.804)	12.081	0.0005	0.612 (0.548–0.675)	–	–	–
Hormones (yes)	2.235 (1.357–3.681)	11.371	0.0007	0.586 (0.532–0.640)	–	–	–
Overall high grade (%)	1.720 (1.236–2.392)	10.181	0.0014	0.611 (0.547–0.675)	1.507 (1.064–2.134)	5.269	0.0217
Log(1 + PSA)	1.555 (1.123–2.153)	7.139	0.0075	0.600 (0.535–0.664)	–	–	–
Age	1.078 (0.804–1.444)	0.254	0.6142	0.509 (0.445–0.573)	–	–	–
					LR test (d.f., *P*‐value) = 28.675 (3, 2.62 × 10^−6^)		
					Harrell’s C‐index (95% CI) = 0.677 (0.613–0.741)		

IQR HRs were used for continuous predictors, and HRs were used for categorical predictors.

C‐index, Harrell’s C‐index; CI, confidence interval; HR, hazard ratio; IQR, interquartile range; LR$\chi_{1}^{2}$, likelihood ratio chi‐square test (d.f. = 1); PSA, prostate‐specific antigen.

**Figure 3:**
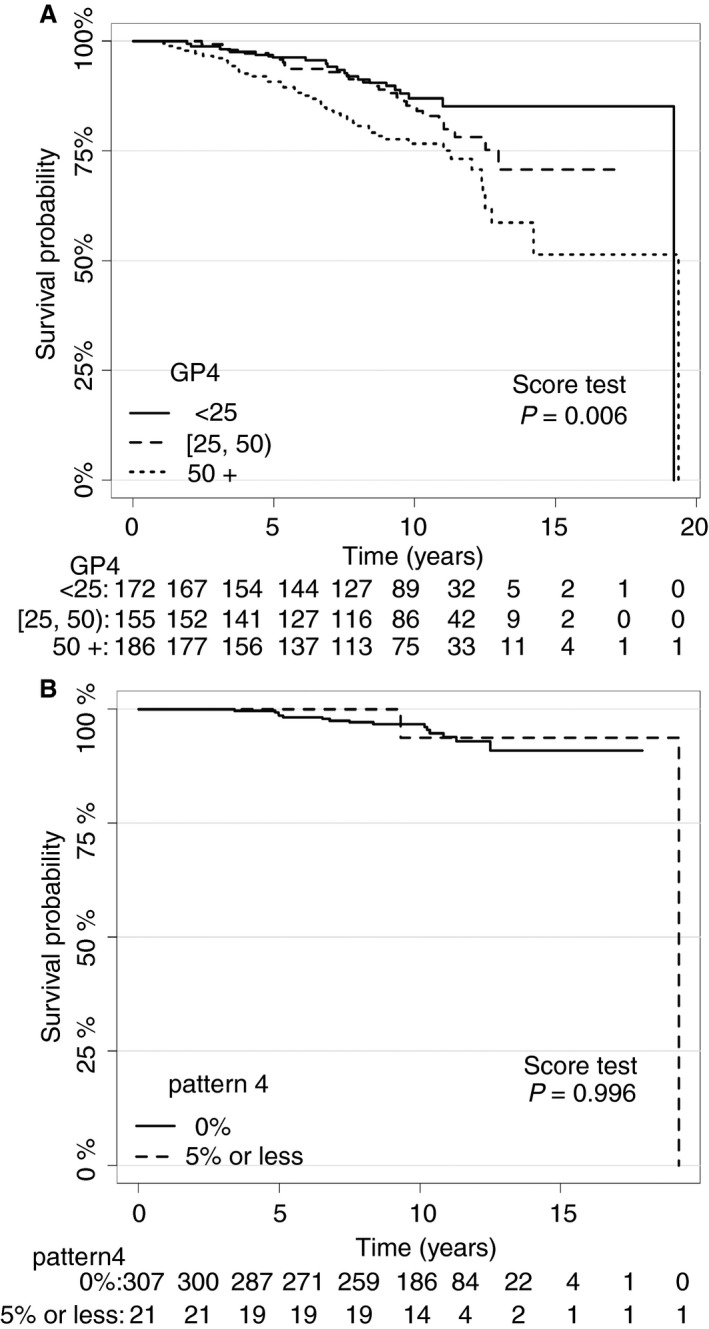
**A,** Kaplan–Meier (KM) curves showing a clear separation of Grade Groups 2 and 3 into three separate groups based on practical percentage values. **B,** KM curve showing no separation between Grade Group 1 and Grade Group 2 with a cut‐off of ≤5% of Gleason pattern 4 disease.

When cut‐offs of <25%, 25% to <50% and >50% Gleason pattern 4 disease were used, three separate cohorts were formed (Figure 3A). The use of an alternative ≤5% cut‐off for Gleason pattern 4 disease in GG2 resulted in comparable survival to the GG1 cohort (Figure 3B).

## Discussion

Assessment of the percentage of Gleason patterns 4 and 5 in prostate biopsies has huge potential to assist in decision‐making prior to patient treatment. Previous studies have shown the power of this assessment to predict biochemical relapse after radical prostatectomy, and also prostate cancer death after TURP. However, none of these studies addressed the most pressing questions for clinicians and patients immediately after prostate cancer diagnosis on biopsy: whether to treat the patient with a radical method or to allow active surveillance. Active surveillance is usually limited to GS 3 + 3 = 6 (GG1) patients; however, GS 3 + 4 = 7 (GG2) patients also have a very good prognosis. Our study shows that this is especially true of those with minimal Gleason pattern 4 disease.

There exists, and will continue to exist, intraobserver error in Gleason scoring^15^, ^16^; a relatively large population of GS 3 + 4 = 7 patients (GG2) are often treated radically, when many have a very good outcome with active surveillance.

It is often difficult for pathologists to give an opinion on reporting very minor small areas of Gleason pattern 4. Borderline decisions may mean that the change from GG1 to GG2 may lead to entirely different treatments. Reporting the percentage of HG disease allows the clear differentiation of cases in which only minor elements of HG disease are identified from those cases that possibly verge on a GS of 4 + 3 = 7 (GG3), which has been shown to indicate a much worse prognosis. Such reporting will enable trials and investigations into the active surveillance of a selected group of men with GG2 disease, below a certain percentage threshold.

The methodology to be used for providing a percentage of HG disease is contentious. The use of an overall or worst GS has previously been extensively debated in the literature.^19^, ^20^, ^21^ There is great variability in how GS is assigned in different centres and how it is interpreted by clinicians.^22^ Some have advocated assigning a GS to every core and giving no overall score for the case. Other pathologists give a GS per submitted specimen pot, which might include more than one core.^15^

In this study, we uniquely assessed both global and worst core methods of assigning percentage of HG disease in relation to prostate cancer death in a large univariable and multivariable analysis with other prognostic pathological parameters.

This has shown, similarly to our findings in the same cohort on GGs,^4^ that both methodologies provide highly significant results, and that the overall percentage of HG disease outperforms the percentage in the worst core both in the overall cohort and in GG2 and GG3, where it is likely to be of more clinical significance. A variety of methods have been suggested for the calculation of the percentage of HG disease in routine reports^10^, ^23^, ^24^ and on the practical problems that it entails.^25^

The strengths of this study include the large sample size and detailed nature of the centralised pathological review and long‐term follow‐up. In some series, it is unclear whether individual cores have been separately graded, especially when they are processed within one cassette or slide.

The weaknesses of the study include its retrospective nature, and the criticism that prostate cancer is now treated differently than it was 10 years ago. The majority of the cohort are from sextant biopsies, and performance of these is not contemporary practice. This is an axiomatic weakness of retrospective prostate studies, to allow time to utilise prostate cancer death as an outcome. Even in large prospective trials such as PROTECT,^26^ the biopsy methods are now considered to have resulted in undersampling of potential disease.

In this study, we did not address the significance of different patterns of HG disease,^27^, ^28^, ^29^ but acknowledge that this is of great interest, and hope to address it in this cohort in a future study.

We have validated, for the first time, the considerable power of assessments of the percentage of HG disease and Gleason pattern 4 to predict prostate cancer death. We recommend that assessments of HG disease be made mandatory in datasets, as they may significantly impact on treatment options after biopsy. The preferred methods of assessment will need consensus in the international pathological community in order to optimise treatment choices.

## Conflicts of interest

The work was partly funded by Myriad Genetics in the form of a research grant.

## Author contributions

D. M. Berney, L. Beltran and G. Soosay reviewed the pathological material. A. Ahmed and J. Murphy performed the statistical analysis. H. Møller led the retrieval of the outcome data. H. Sandu collated the TAPG database. P. Scardino helped to envisage the TAPG study. D. M. Berney wrote the article and guided this particular TAPG paper. J. Cuzick leads the TAPG consortium and initiated the whole TAPG study. All authors reviewed and commented upon the paper.
